# Supplementary material for: Genome-Wide Identification and Characterization of Xyloglucan Endotransglycosylase/Hydrolase in Ananas comosus during Development
Source: Genes (Basel). 2019 Jul 16;10(7):537. doi: 10.3390/genes10070537 (PMC6678617; doi:10.3390/genes10070537)
Supplement: Supplementary file 1 [file genes-10-00537-s001.zip › Supplementfiles/File 5.docx]

| **Table S1. Primer sequence used in qRT-PCR.** | |
| --- | --- |
| **Gene member** | **Primer sequence** |
| *Ac(XTH)XTH23F* | GGGATTCATCTCCTCCGATTAC |
| *Ac(XTH)XTH23R* | CATCGCCATTCGATAGGTAGAA |
| *Ac(XTH)XTH18F* | CCAAACCCATGTCCCTCTATG |
| *Ac(XTH)XTH18R* | CGCAACGAACGGAGAGTATT |
| *Ac(XTH)XTH8F* | GTGCAGACGAACCTGTACATA |
| *Ac(XTH)XTH8R* | GTATGCTGTAGGAGTGGAAGTC |
| *Ac(XTH)XTH6F* | GGCCGACTTCCACACTTATT |
| *Ac(XTH)XTH6R* | CTCCTTGTTTGGGAACATCCT |
| *Ac(XTH)XTH2F* | GACAGGCCAGCCTTACATATT |
| *Ac(XTH)XTH2R* | TAGCACAGAGTAGGAGTGGTAG |
| *Ac(XTH)XTH5F* | GATGAAGTGGGTGCAGGATAA |
| *Ac(XTH)XTH5R* | AGAGAGAGAGCGAGAAGAGAAG |
| *Ac(XTH)XTH15F* | GCTACCACACATACACCATCTT |
| *Ac(XTH)XTH15R* | CCTTGCTGTTCCGGTTCTT |
| *Ac(XTH)XTH16F* | GGAGCAGCAGCAGAATCATA |
| *Ac(XTH)XTH16R* | CATGGGCTTGGATGGAAATTG |
| *Ac(XTH)XTH11F* | AATGTTAGCGGAGAGCCTTAC |
| *Ac(XTH)XTH11R* | TGGGATCAAACCACAAGTAGAA |
| *Ac(XTH)XTH20F* | ACCTACTCCTTCTGCTACGA |
| *Ac(XTH)XTH20R* | ATGCCCGGTCTCCTTAAATC |
| *Ac(XTH)XTH13F* | GTGTCCTTCCACAACTACTCTATC |
| *Ac(XTH)XTH13R* | CCATCTTCTCGCTGTTCCTAAA |
| *Actin2-F* | GTATGGAAGCTGCGGGTATT |
| *Actin2-R* | CACCACTGAGCACGATGTTA |
