# Supplementary material for: Genome-Wide Identification and Characterization of Xyloglucan Endotransglycosylase/Hydrolase in Ananas comosus during Development
Source: Genes (Basel). 2019 Jul 16;10(7):537. doi: 10.3390/genes10070537 (PMC6678617; doi:10.3390/genes10070537)
Supplement: Supplementary file 1 [file genes-10-00537-s001.zip › Supplementfiles/File 6.docx]

| **Table S2. The position information of XTHs in F153 and MD2.** | | | | | |
| --- | --- | --- | --- | --- | --- |
| Name | Transcript ID | Accession | Start | End | Strain |
| Ac(F153)XTH1 | XP_020091206.1 | NC_033626.1 | 12567167 | 12569266 | - |
| Ac(F153)XTH2 | XP_020111283.1 | NC_033641.1 | 113827 | 116141 | - |
| Ac(F153)XTH3 | XP_020087864.1 | NC_033625.1 | 4883793 | 4886925 | + |
| Ac(F153)XTH4 | XP_020102739.1 | NC_033634.1 | 1534703 | 1537395 | + |
| Ac(F153)XTH5 | XP_020102738.1 | NC_033634.1 | 1534662 | 1537395 | + |
| Ac(F153)XTH6 | XP_020109096.1 | NC_033639.1 | 10644860 | 10646399 | + |
| Ac(F153)XTH7 | XP_020084286.1 | NC_033623.1 | 728574 | 730962 | - |
| Ac(F153)XTH8 | XP_020104828.1 | NC_033635.1 | 10932826 | 10934596 | + |
| Ac(F153)XTH9 | XP_020089756.1 | NC_033626.1 | 2941439 | 2942700 | + |
| Ac(F153)XTH10 | XP_020090359.1 | NC_033626.1 | 2921434 | 2922731 | - |
| Ac(F153)XTH11 | XP_020110218.1 | NC_033640.1 | 7024227 | 7026493 | + |
| Ac(F153)XTH12 | XP_020102740.1 | NC_033634.1 | 1528550 | 1529903 | + |
| Ac(F153)XTH13 | XP_020091231.1 | NC_033626.1 | 2776350 | 2777972 | + |
| Ac(F153)XTH14 | XP_020090869.1 | NC_033626.1 | 3471272 | 3474785 | + |
| Ac(F153)XTH15 | XP_020097886.1 | NC_033630.1 | 4268865 | 4271189 | - |
| Ac(F153)XTH16 | XP_020100605.1 | NC_033632.1 | 2683422 | 2685571 | + |
| Ac(F153)XTH17 | XP_020106929.1 | NC_033622.1 | 14874640 | 14876892 | + |
| Ac(F153)XTH18 | XP_020104936.1 | NC_033635.1 | 10378544 | 10382172 | + |
| Ac(F153)XTH19 | XP_020092864.1 | NC_033627.1 | 12958700 | 12960981 | - |
| Ac(F153)XTH20 | XP_020094226.1 | NC_033628.1 | 13435909 | 13439155 | - |
| Ac(F153)XTH21 | XP_020085280.1 | NC_033623.1 | 16205413 | 16208261 | - |
| Ac(F153)XTH22 | XP_020085278.1 | NC_033623.1 | 16137609 | 16142138 | - |
| Ac(F153)XTH23 | XP_020085279.1 | NC_033623.1 | 16137609 | 16142141 | - |
| Ac(F153)XTH24 | XP_020112380.1 | NC_033642.1 | 9264816 | 9266773 | - |
| Ac(MD2)XTH1 | OAY79161.1 | LSRQ01001111.1 | 561367 | 562480 | + |
| Ac(MD2)XTH2 | OAY64709.1 | LSRQ01007698.1 | 92617 | 95279 | + |
| Ac(MD2)XTH3 | OAY72845.1 | LSRQ01002960.1 | 1842 | 3632 | + |
| Ac(MD2)XTH4 | OAY65283.1 | LSRQ01007065.1 | 10599 | 12462 | - |
| Ac(MD2)XTH5 | OAY76125.1 | LSRQ01001906.1 | 15489 | 27739 | + |
| Ac(MD2)XTH6 | OAY78767.1 | LSRQ01001221.1 | 108537 | 110823 | - |
| Ac(MD2)XTH7 | OAY70160.1 | LSRQ01004034.1 | 11004 | 12231 | + |
| Ac(MD2)XTH8 | OAY63484.1 | LSRQ01008337.1 | 281105 | 282334 | - |
| Ac(MD2)XTH9 | OAY62696.1 | LSRQ01008439.1 | 94482 | 95548 | - |
| Ac(MD2)XTH10 | OAY67076.1 | LSRQ01005732.1 | 94222 | 95306 | - |
| Ac(MD2)XTH11 | OAY62698.1 | LSRQ01008439.1 | 11668 | 14647 | + |
| Ac(MD2)XTH12 | OAY79036.1 | LSRQ01001144.1 | 273745 | 275440 | - |
| Ac(MD2)XTH13 | OAY76653.1 | LSRQ01001756.1 | 9302 | 11160 | - |
| Ac(MD2)XTH14 | OAY71418.1 | LSRQ01003478.1 | 13728 | 15607 | - |
| Ac(MD2)XTH15 | OAY73488.1 | LSRQ01002677.1 | 198641 | 199968 | + |
| Ac(MD2)XTH16 | OAY70295.1 | LSRQ01003966.1 | 1772 | 5023 | - |
| Ac(MD2)XTH17 | OAY81259.1 | LSRQ01000699.1 | 148018 | 151157 | + |
| Ac(MD2)XTH18 | OAY66122.1 | LSRQ01006443.1 | 163169 | 164837 | - |
| Ac(MD2)XTH19 | OAY83621.1 | LSRQ01000330.1 | 28014 | 29843 | + |
| Ac(MD2)XTH20 | OAY70631.1 | LSRQ01003822.1 | 15325 | 17146 | - |
| Ac(MD2)XTH21 | OAY84325.1 | LSRQ01000231.1 | 93050 | 96648 | + |
| Ac(MD2)XTH22 | OAY70279.1 | LSRQ01003976.1 | 29037 | 31464 | + |
| Ac(MD2)XTH23 | OAY81925.1 | LSRQ01000568.1 | 20725 | 24005 | - |
| Ac(MD2)XTH24 | OAY65080.1 | LSRQ01007309.1 | 1313 | 4591 | + |
