# Supplementary material for: Genome-Wide Identification and Characterization of Xyloglucan Endotransglycosylase/Hydrolase in Ananas comosus during Development
Source: Genes (Basel). 2019 Jul 16;10(7):537. doi: 10.3390/genes10070537 (PMC6678617; doi:10.3390/genes10070537)
Supplement: Supplementary file 1 [file genes-10-00537-s001.zip › Supplementfiles/File 7.docx]

| **Table S3. The ratio of Ka/Ks in XTH gene pairs based on P-value < 0.05.** | | | | | |
| --- | --- | --- | --- | --- | --- |
| **Pairs** |  | **Ks** | **Ka/Ks** | **P-value** | **Divergence-Time (Mya)** |
| *Ac(F153)XTH24/Ac(MD2)XTH22* | 0.587727 | 1.15969 | 0.506799 | 7.13E-06 | 95.056557 |
| *Ac(F153)XTH24/Ac(MD2)XTH21* | 0.58637 | 1.16789 | 0.502078 | 3.26E-06 | 95.728689 |
| *Ac(F153)XTH20/Ac(F153)XTH24* | 0.604765 | 1.24953 | 0.483994 | 1.99E-06 | 102.42049 |
| *Ac(F153)XTH24/Ac(MD2)XTH23* | 0.604516 | 1.25233 | 0.482711 | 1.99E-06 | 102.65 |
| *Ac(F153)XTH22/Ac(F153)XTH24* | 0.571966 | 1.19795 | 0.477456 | 9.44E-07 | 98.192623 |
| *Ac(F153)XTH23/Ac(F153)XTH24* | 0.571966 | 1.19795 | 0.477456 | 9.44E-07 | 98.192623 |
| *Ac(F153)XTH24/Ac(MD2)XTH24* | 0.660851 | 1.40108 | 0.471672 | 9.26E-07 | 114.84262 |
| *Ac(F153)XTH21/Ac(F153)XTH24* | 0.569076 | 1.24323 | 0.457738 | 2.69E-07 | 101.9041 |
| *Ac(F153)XTH10/Ac(MD2)XTH15* | 0.489052 | 1.07385 | 0.455421 | 5.00E-07 | 88.020492 |
| *Ac(F153)XTH16/Ac(MD2)XTH9* | 0.454151 | 1.0109 | 0.449257 | 4.58E-07 | 82.860656 |
| *Ac(F153)XTH10/Ac(F153)XTH16* | 0.489052 | 1.09743 | 0.445633 | 3.13E-07 | 89.953279 |
| *Ac(MD2)XTH9/Ac(MD2)XTH15* | 0.454151 | 1.03392 | 0.439253 | 3.02E-07 | 84.747541 |
| *Ac(MD2)XTH10/Ac(MD2)XTH15* | 0.454151 | 1.03392 | 0.439253 | 3.02E-07 | 84.747541 |
| *Ac(F153)XTH16/Ac(MD2)XTH10* | 0.454151 | 1.05767 | 0.429389 | 1.09E-07 | 86.694262 |
| *Ac(F153)XTH9/Ac(MD2)XTH15* | 0.474608 | 1.1112 | 0.427114 | 4.89E-08 | 91.081967 |
| *Ac(F153)XTH9/Ac(F153)XTH16* | 0.474608 | 1.1359 | 0.417824 | 2.78E-08 | 93.106557 |
| *Ac(F153)XTH10/Ac(F153)XTH14* | 0.375418 | 0.904285 | 0.415154 | 1.41E-08 | 74.121721 |
| *Ac(F153)XTH12/Ac(F153)XTH14* | 0.354814 | 0.862331 | 0.41146 | 4.22E-09 | 70.682869 |
| *Ac(F153)XTH10/Ac(MD2)XTH11* | 0.370063 | 0.904162 | 0.409288 | 8.01E-09 | 74.111639 |
| *Ac(F153)XTH19/Ac(F153)XTH24* | 0.568269 | 1.39049 | 0.408683 | 6.58E-09 | 113.97459 |
| *Ac(F153)XTH24/Ac(MD2)XTH19* | 0.48177 | 1.19542 | 0.403015 | 1.04E-07 | 97.985246 |
| *Ac(MD2)XTH10/Ac(MD2)XTH11* | 0.351009 | 0.898664 | 0.39059 | 4.18E-09 | 73.660984 |
| *Ac(F153)XTH12/Ac(MD2)XTH11* | 0.334417 | 0.85626 | 0.390556 | 1.49E-09 | 70.185246 |
| *Ac(F153)XTH24/Ac(MD2)XTH20* | 0.486177 | 1.25473 | 0.387476 | 2.15E-08 | 102.84672 |
| *Ac(F153)XTH13/Ac(MD2)XTH11* | 0.327374 | 0.845784 | 0.387066 | 9.10E-10 | 69.326557 |
| *Ac(MD2)XTH9/Ac(MD2)XTH11* | 0.351009 | 0.917789 | 0.382451 | 2.22E-09 | 75.228607 |
| *Ac(F153)XTH14/Ac(MD2)XTH10* | 0.35056 | 0.921024 | 0.38062 | 1.26E-09 | 75.49377 |
| *Ac(F153)XTH14/Ac(MD2)XTH9* | 0.35056 | 0.940735 | 0.372645 | 3.76E-10 | 77.109426 |
| *Ac(F153)XTH19/Ac(F153)XTH22* | 0.277346 | 0.750409 | 0.369593 | 2.46E-12 | 61.508934 |
| *Ac(F153)XTH19/Ac(F153)XTH23* | 0.277346 | 0.750409 | 0.369593 | 2.46E-12 | 61.508934 |
| *Ac(F153)XTH19/Ac(MD2)XTH22* | 0.280089 | 0.757893 | 0.369563 | 1.41E-12 | 62.122377 |
| *Ac(MD2)XTH6/Ac(MD2)XTH7* | 0.386221 | 1.04524 | 0.369505 | 3.65E-11 | 85.67541 |
| *Ac(F153)XTH7/Ac(MD2)XTH4* | 0.49919 | 1.35322 | 0.36889 | 1.15E-10 | 110.91967 |
| *Ac(F153)XTH2/Ac(F153)XTH7* | 0.49919 | 1.35322 | 0.36889 | 1.15E-10 | 110.91967 |
| *Ac(F153)XTH19/Ac(MD2)XTH21* | 0.280205 | 0.76791 | 0.364893 | 7.94E-13 | 62.943443 |
| *Ac(MD2)XTH6/Ac(MD2)XTH8* | 0.383457 | 1.05106 | 0.364828 | 1.81E-11 | 86.152459 |
| *Ac(F153)XTH6/Ac(MD2)XTH6* | 0.383457 | 1.05106 | 0.364828 | 1.81E-11 | 86.152459 |
| *Ac(MD2)XTH4/Ac(MD2)XTH6* | 0.499523 | 1.37902 | 0.362232 | 3.66E-11 | 113.03443 |
| *Ac(F153)XTH2/Ac(MD2)XTH6* | 0.499523 | 1.37902 | 0.362232 | 3.66E-11 | 113.03443 |
| *Ac(F153)XTH12/Ac(MD2)XTH15* | 0.502967 | 1.40492 | 0.358004 | 5.26E-11 | 115.15738 |
| *Ac(F153)XTH9/Ac(MD2)XTH11* | 0.355241 | 0.992452 | 0.357942 | 3.57E-11 | 81.348525 |
| *Ac(F153)XTH9/Ac(F153)XTH14* | 0.36439 | 1.02073 | 0.356991 | 3.19E-11 | 83.666393 |
| *Ac(MD2)XTH5/Ac(MD2)XTH8* | 0.489692 | 1.38314 | 0.354045 | 5.47E-12 | 113.37213 |
| *Ac(F153)XTH6/Ac(MD2)XTH5* | 0.489692 | 1.38314 | 0.354045 | 5.47E-12 | 113.37213 |
| *Ac(F153)XTH19/Ac(F153)XTH21* | 0.274611 | 0.777051 | 0.353401 | 2.12E-13 | 63.692705 |
| *Ac(MD2)XTH20/Ac(MD2)XTH21* | 0.24794 | 0.701639 | 0.353373 | 8.91E-11 | 57.511393 |
| *Ac(MD2)XTH19/Ac(MD2)XTH21* | 0.250953 | 0.718284 | 0.349378 | 3.79E-11 | 58.875738 |
| *Ac(F153)XTH12/Ac(F153)XTH16* | 0.502967 | 1.43992 | 0.349302 | 2.77E-11 | 118.02623 |
| *Ac(F153)XTH12/Ac(F153)XTH15* | 0.443464 | 1.27132 | 0.348823 | 8.94E-12 | 104.20656 |
| *Ac(F153)XTH13/Ac(F153)XTH14* | 0.311762 | 0.902198 | 0.345558 | 1.07E-11 | 73.950656 |
| *Ac(F153)XTH22/Ac(MD2)XTH20* | 0.244566 | 0.708447 | 0.345214 | 2.13E-11 | 58.069426 |
| *Ac(F153)XTH23/Ac(MD2)XTH20* | 0.244566 | 0.708447 | 0.345214 | 2.13E-11 | 58.069426 |
| *Ac(MD2)XTH3/Ac(MD2)XTH7* | 0.449918 | 1.30391 | 0.345052 | 2.12E-11 | 106.87787 |
| *Ac(MD2)XTH20/Ac(MD2)XTH22* | 0.245683 | 0.715068 | 0.343579 | 2.51E-11 | 58.612131 |
| *Ac(MD2)XTH5/Ac(MD2)XTH7* | 0.483026 | 1.406 | 0.343546 | 1.50E-12 | 115.2459 |
| *Ac(MD2)XTH3/Ac(MD2)XTH8* | 0.449231 | 1.31327 | 0.34207 | 1.12E-11 | 107.64508 |
| *Ac(F153)XTH6/Ac(MD2)XTH3* | 0.449231 | 1.31327 | 0.34207 | 1.12E-11 | 107.64508 |
| *Ac(F153)XTH22/Ac(MD2)XTH19* | 0.247556 | 0.725195 | 0.341365 | 1.60E-11 | 59.442213 |
| *Ac(F153)XTH23/Ac(MD2)XTH19* | 0.247556 | 0.725195 | 0.341365 | 1.60E-11 | 59.442213 |
| *Ac(F153)XTH7/Ac(MD2)XTH3* | 0.477428 | 1.40131 | 0.340701 | 1.34E-11 | 114.86148 |
| *Ac(MD2)XTH19/Ac(MD2)XTH22* | 0.24868 | 0.731911 | 0.339768 | 1.65E-11 | 59.992705 |
| *Ac(MD2)XTH4/Ac(MD2)XTH7* | 0.474898 | 1.40608 | 0.337746 | 1.23E-12 | 115.25246 |
| *Ac(F153)XTH2/Ac(MD2)XTH7* | 0.474898 | 1.40608 | 0.337746 | 1.23E-12 | 115.25246 |
| *Ac(F153)XTH7/Ac(MD2)XTH2* | 0.452727 | 1.34694 | 0.336114 | 5.31E-12 | 110.40492 |
| *Ac(F153)XTH4/Ac(MD2)XTH5* | 0.0558479 | 0.166419 | 0.335585 | 6.33E-05 | 13.640902 |
| *Ac(F153)XTH10/Ac(F153)XTH15* | 0.440469 | 1.31656 | 0.334561 | 5.13E-12 | 107.91475 |
| *Ac(MD2)XTH3/Ac(MD2)XTH6* | 0.477758 | 1.43054 | 0.333969 | 7.67E-12 | 117.25738 |
| *Ac(MD2)XTH7/Ac(MD2)XTH8* | 0.0215163 | 0.0644584 | 0.333802 | 0.0033582 | 5.2834754 |
| *Ac(F153)XTH6/Ac(MD2)XTH7* | 0.0215163 | 0.0644584 | 0.333802 | 0.0033582 | 5.2834754 |
| *Ac(MD2)XTH4/Ac(MD2)XTH8* | 0.476079 | 1.42702 | 0.333617 | 6.80E-13 | 116.96885 |
| *Ac(F153)XTH6/Ac(MD2)XTH4* | 0.476079 | 1.42702 | 0.333617 | 6.80E-13 | 116.96885 |
| *Ac(F153)XTH2/Ac(MD2)XTH8* | 0.476079 | 1.42702 | 0.333617 | 6.80E-13 | 116.96885 |
| *Ac(F153)XTH2/Ac(F153)XTH6* | 0.476079 | 1.42702 | 0.333617 | 6.80E-13 | 116.96885 |
| *Ac(F153)XTH13/Ac(MD2)XTH15* | 0.487268 | 1.46403 | 0.332826 | 7.13E-12 | 120.00246 |
| *Ac(F153)XTH3/Ac(MD2)XTH7* | 0.473241 | 1.4321 | 0.330452 | 5.93E-13 | 117.38525 |
| *Ac(F153)XTH7/Ac(MD2)XTH7* | 0.362195 | 1.10598 | 0.327488 | 2.17E-13 | 90.654098 |
| *Ac(F153)XTH21/Ac(MD2)XTH20* | 0.241385 | 0.739918 | 0.326231 | 3.01E-12 | 60.649016 |
| *Ac(F153)XTH3/Ac(MD2)XTH8* | 0.469832 | 1.44239 | 0.325731 | 2.95E-13 | 118.22869 |
| *Ac(F153)XTH3/Ac(F153)XTH6* | 0.469832 | 1.44239 | 0.325731 | 2.95E-13 | 118.22869 |
| *Ac(F153)XTH13/Ac(F153)XTH16* | 0.487268 | 1.50346 | 0.324097 | 3.43E-12 | 123.23443 |
| *Ac(F153)XTH15/Ac(MD2)XTH10* | 0.398937 | 1.23129 | 0.324 | 2.16E-12 | 100.92541 |
| *Ac(F153)XTH9/Ac(F153)XTH15* | 0.434294 | 1.34467 | 0.322973 | 8.08E-13 | 110.21885 |
| *Ac(F153)XTH21/Ac(MD2)XTH19* | 0.244355 | 0.757095 | 0.322754 | 1.19E-12 | 62.056967 |
| *Ac(MD2)XTH2/Ac(MD2)XTH7* | 0.428491 | 1.33233 | 0.321609 | 1.13E-12 | 109.20738 |
| *Ac(MD2)XTH2/Ac(MD2)XTH6* | 0.454805 | 1.42099 | 0.320062 | 1.68E-12 | 116.47459 |
| *Ac(MD2)XTH2/Ac(MD2)XTH8* | 0.425024 | 1.34216 | 0.316671 | 5.60E-13 | 110.01311 |
| *Ac(F153)XTH6/Ac(MD2)XTH2* | 0.425024 | 1.34216 | 0.316671 | 5.60E-13 | 110.01311 |
| *Ac(F153)XTH7/Ac(MD2)XTH8* | 0.359491 | 1.13548 | 0.316598 | 5.19E-14 | 93.072131 |
| *Ac(F153)XTH6/Ac(F153)XTH7* | 0.359491 | 1.13548 | 0.316598 | 5.19E-14 | 93.072131 |
| *Ac(F153)XTH15/Ac(MD2)XTH9* | 0.398937 | 1.26218 | 0.316068 | 1.15E-12 | 103.45738 |
| *Ac(F153)XTH9/Ac(F153)XTH12* | 0.269664 | 0.872116 | 0.309206 | 1.61E-13 | 71.484918 |
| *Ac(F153)XTH10/Ac(F153)XTH12* | 0.267414 | 0.871458 | 0.306858 | 2.55E-13 | 71.430984 |
| *Ac(MD2)XTH16/Ac(MD2)XTH22* | 0.39764 | 1.32897 | 0.299209 | 6.52E-17 | 108.93197 |
| *Ac(F153)XTH22/Ac(MD2)XTH16* | 0.395381 | 1.32226 | 0.29902 | 3.47E-17 | 108.38197 |
| *Ac(F153)XTH23/Ac(MD2)XTH16* | 0.395381 | 1.32226 | 0.29902 | 3.47E-17 | 108.38197 |
| *Ac(F153)XTH18/Ac(MD2)XTH22* | 0.388611 | 1.30315 | 0.298208 | 1.56E-17 | 106.81557 |
| *Ac(F153)XTH3/Ac(F153)XTH7* | 0.482707 | 1.62072 | 0.297835 | 4.24E-14 | 132.8459 |
| *Ac(MD2)XTH16/Ac(MD2)XTH21* | 0.398616 | 1.34222 | 0.296982 | 3.79E-17 | 110.01803 |
| *Ac(F153)XTH18/Ac(MD2)XTH21* | 0.389544 | 1.31569 | 0.296075 | 8.11E-18 | 107.84344 |
| *Ac(F153)XTH3/Ac(MD2)XTH6* | 0.478911 | 1.61828 | 0.295939 | 7.58E-15 | 132.6459 |
| *Ac(F153)XTH18/Ac(F153)XTH22* | 0.388707 | 1.32144 | 0.294154 | 8.74E-18 | 108.31475 |
| *Ac(F153)XTH18/Ac(F153)XTH23* | 0.388707 | 1.32144 | 0.294154 | 8.74E-18 | 108.31475 |
| *Ac(MD2)XTH11/Ac(MD2)XTH15* | 0.470991 | 1.6099 | 0.292559 | 1.94E-14 | 131.95902 |
| *Ac(F153)XTH13/Ac(F153)XTH15* | 0.448046 | 1.5328 | 0.292306 | 9.22E-15 | 125.63934 |
| *Ac(F153)XTH20/Ac(MD2)XTH16* | 0.407483 | 1.39574 | 0.291948 | 2.95E-17 | 114.40492 |
| *Ac(F153)XTH12/Ac(MD2)XTH10* | 0.250518 | 0.858421 | 0.291835 | 5.78E-14 | 70.362377 |
| *Ac(F153)XTH15/Ac(MD2)XTH15* | 0.379423 | 1.30372 | 0.29103 | 2.15E-15 | 106.8623 |
| *Ac(F153)XTH19/Ac(MD2)XTH23* | 0.295293 | 1.0201 | 0.289476 | 1.05E-18 | 83.614754 |
| *Ac(MD2)XTH16/Ac(MD2)XTH24* | 0.447561 | 1.56009 | 0.286881 | 6.75E-17 | 127.87623 |
| *Ac(F153)XTH12/Ac(MD2)XTH9* | 0.250518 | 0.876859 | 0.285699 | 2.82E-14 | 71.873689 |
| *Ac(F153)XTH19/Ac(F153)XTH20* | 0.295376 | 1.03483 | 0.285433 | 2.37E-19 | 84.822131 |
| *Ac(MD2)XTH16/Ac(MD2)XTH23* | 0.407353 | 1.42811 | 0.285239 | 7.17E-18 | 117.0582 |
| *Ac(F153)XTH22/Ac(MD2)XTH17* | 0.43462 | 1.52646 | 0.284725 | 8.91E-18 | 125.11967 |
| *Ac(F153)XTH23/Ac(MD2)XTH17* | 0.43462 | 1.52646 | 0.284725 | 8.91E-18 | 125.11967 |
| *Ac(F153)XTH12/Ac(F153)XTH13* | 0.288895 | 1.01501 | 0.284623 | 1.01E-15 | 83.197541 |
| *Ac(MD2)XTH17/Ac(MD2)XTH21* | 0.438817 | 1.54176 | 0.284621 | 8.98E-18 | 126.37377 |
| *Ac(MD2)XTH17/Ac(MD2)XTH22* | 0.436949 | 1.53603 | 0.284467 | 8.59E-18 | 125.9041 |
| *Ac(F153)XTH16/Ac(MD2)XTH11* | 0.470991 | 1.6564 | 0.284346 | 9.64E-15 | 135.77049 |
| *Ac(F153)XTH15/Ac(F153)XTH16* | 0.379423 | 1.33446 | 0.284327 | 1.07E-15 | 109.38197 |
| *Ac(F153)XTH21/Ac(MD2)XTH16* | 0.392093 | 1.38303 | 0.283503 | 1.99E-18 | 113.36311 |
| *Ac(F153)XTH19/Ac(MD2)XTH24* | 0.32543 | 1.15873 | 0.280849 | 1.47E-18 | 94.977869 |
| *Ac(F153)XTH14/Ac(MD2)XTH15* | 0.469403 | 1.67761 | 0.279804 | 2.50E-15 | 137.50902 |
| *Ac(F153)XTH18/Ac(F153)XTH24* | 0.538526 | 1.92505 | 0.279746 | 1.90E-14 | 157.79098 |
| *Ac(F153)XTH18/Ac(F153)XTH21* | 0.38554 | 1.38071 | 0.279233 | 2.48E-19 | 113.17295 |
| *Ac(F153)XTH24/Ac(MD2)XTH16* | 0.530209 | 1.90573 | 0.278219 | 1.32E-14 | 156.20738 |
| *Ac(F153)XTH18/Ac(F153)XTH20* | 0.402483 | 1.44749 | 0.278056 | 5.70E-19 | 118.64672 |
| *Ac(F153)XTH20/Ac(MD2)XTH17* | 0.448938 | 1.64139 | 0.273511 | 3.07E-18 | 134.54016 |
| *Ac(MD2)XTH3/Ac(MD2)XTH4* | 0.0078067 | 0.0285993 | 0.272967 | 0.0441629 | 2.3442049 |
| *Ac(F153)XTH2/Ac(MD2)XTH3* | 0.0078067 | 0.0285993 | 0.272967 | 0.0441629 | 2.3442049 |
| *Ac(F153)XTH18/Ac(MD2)XTH24* | 0.438572 | 1.6132 | 0.271864 | 2.62E-18 | 132.22951 |
| *Ac(F153)XTH18/Ac(MD2)XTH23* | 0.402358 | 1.48137 | 0.271612 | 1.20E-19 | 121.42377 |
| *Ac(F153)XTH14/Ac(F153)XTH16* | 0.469403 | 1.72857 | 0.271556 | 1.26E-15 | 141.68607 |
| *Ac(F153)XTH21/Ac(MD2)XTH17* | 0.431191 | 1.60709 | 0.268305 | 5.73E-19 | 131.72869 |
| *Ac(MD2)XTH17/Ac(MD2)XTH23* | 0.448792 | 1.68641 | 0.266123 | 7.30E-19 | 138.23033 |
| *Ac(F153)XTH15/Ac(MD2)XTH11* | 0.436167 | 1.63975 | 0.265996 | 3.71E-16 | 134.40574 |
| *Ac(MD2)XTH19/Ac(MD2)XTH23* | 0.249003 | 0.941093 | 0.264589 | 2.16E-17 | 77.13877 |
| *Ac(F153)XTH19/Ac(MD2)XTH17* | 0.442851 | 1.68268 | 0.263183 | 1.49E-19 | 137.92459 |
| *Ac(MD2)XTH17/Ac(MD2)XTH24* | 0.530032 | 2.01502 | 0.26304 | 6.00E-17 | 165.16557 |
| *Ac(F153)XTH20/Ac(MD2)XTH19* | 0.249085 | 0.957614 | 0.26011 | 1.05E-17 | 78.492951 |
| *Ac(MD2)XTH19/Ac(MD2)XTH24* | 0.290889 | 1.12096 | 0.2595 | 1.80E-16 | 91.881967 |
| *Ac(MD2)XTH21/Ac(MD2)XTH24* | 0.203535 | 0.796373 | 0.255577 | 3.47E-19 | 65.276475 |
| *Ac(MD2)XTH20/Ac(MD2)XTH23* | 0.242304 | 0.959335 | 0.252575 | 3.18E-18 | 78.634016 |
| *Ac(MD2)XTH22/Ac(MD2)XTH24* | 0.203761 | 0.808988 | 0.251872 | 1.33E-19 | 66.310492 |
| *Ac(F153)XTH20/Ac(MD2)XTH20* | 0.242383 | 0.976252 | 0.24828 | 6.17E-19 | 80.020656 |
| *Ac(F153)XTH18/Ac(F153)XTH19* | 0.36005 | 1.45097 | 0.248145 | 1.39E-22 | 118.93197 |
| *Ac(F153)XTH10/Ac(F153)XTH13* | 0.285979 | 1.16931 | 0.244572 | 9.93E-19 | 95.845082 |
| *Ac(MD2)XTH21/Ac(MD2)XTH23* | 0.168293 | 0.692272 | 0.243103 | 1.73E-20 | 56.743607 |
| *Ac(F153)XTH9/Ac(F153)XTH13* | 0.288225 | 1.18833 | 0.242546 | 2.53E-18 | 97.404098 |
| *Ac(F153)XTH5/Ac(MD2)XTH8* | 0.419608 | 1.73196 | 0.242273 | 1.96E-18 | 141.96393 |
| *Ac(F153)XTH5/Ac(F153)XTH6* | 0.419608 | 1.73196 | 0.242273 | 1.96E-18 | 141.96393 |
| *Ac(F153)XTH5/Ac(MD2)XTH7* | 0.42832 | 1.76838 | 0.242211 | 2.38E-18 | 144.94918 |
| *Ac(MD2)XTH20/Ac(MD2)XTH24* | 0.281751 | 1.17179 | 0.240445 | 1.57E-18 | 96.048361 |
| *Ac(F153)XTH19/Ac(MD2)XTH16* | 0.366962 | 1.53507 | 0.239052 | 1.10E-22 | 125.82541 |
| *Ac(MD2)XTH22/Ac(MD2)XTH23* | 0.168229 | 0.704164 | 0.238906 | 7.58E-21 | 57.718361 |
| *Ac(F153)XTH20/Ac(MD2)XTH21* | 0.167151 | 0.699985 | 0.238793 | 7.48E-21 | 57.37582 |
| *Ac(F153)XTH14/Ac(F153)XTH15* | 0.430833 | 1.8172 | 0.237086 | 8.25E-18 | 148.95082 |
| *Ac(F153)XTH13/Ac(MD2)XTH10* | 0.267169 | 1.13489 | 0.235415 | 3.98E-18 | 93.02377 |
| *Ac(F153)XTH20/Ac(MD2)XTH22* | 0.167088 | 0.712016 | 0.234669 | 1.03E-21 | 58.361967 |
| *Ac(F153)XTH22/Ac(MD2)XTH24* | 0.199471 | 0.85335 | 0.233751 | 1.72E-21 | 69.946721 |
| *Ac(F153)XTH23/Ac(MD2)XTH24* | 0.199471 | 0.85335 | 0.233751 | 1.72E-21 | 69.946721 |
| *Ac(MD2)XTH12/Ac(MD2)XTH15* | 0.577477 | 2.49508 | 0.231447 | 1.45E-14 | 204.51475 |
| *Ac(F153)XTH16/Ac(MD2)XTH12* | 0.577477 | 2.49508 | 0.231447 | 1.45E-14 | 204.51475 |
| *Ac(F153)XTH21/Ac(MD2)XTH24* | 0.203311 | 0.879428 | 0.231185 | 5.51E-22 | 72.084262 |
| *Ac(F153)XTH13/Ac(MD2)XTH9* | 0.267169 | 1.16216 | 0.229891 | 5.04E-19 | 95.259016 |
| *Ac(F153)XTH5/Ac(MD2)XTH5* | 0.0119433 | 0.0523352 | 0.228207 | 0.0022557 | 4.2897705 |
| *Ac(F153)XTH15/Ac(MD2)XTH12* | 0.615566 | 2.70789 | 0.227323 | 7.34E-14 | 221.9582 |
| *Ac(F153)XTH21/Ac(MD2)XTH21* | 0.0066281 | 0.0292147 | 0.226877 | 0.0118956 | 2.3946475 |
| *Ac(F153)XTH20/Ac(F153)XTH22* | 0.164366 | 0.740651 | 0.221922 | 6.47E-23 | 60.709098 |
| *Ac(F153)XTH20/Ac(F153)XTH23* | 0.164366 | 0.740651 | 0.221922 | 6.47E-23 | 60.709098 |
| *Ac(F153)XTH22/Ac(MD2)XTH23* | 0.164324 | 0.741567 | 0.221591 | 6.13E-23 | 60.78418 |
| *Ac(F153)XTH23/Ac(MD2)XTH23* | 0.164324 | 0.741567 | 0.221591 | 6.13E-23 | 60.78418 |
| *Ac(F153)XTH20/Ac(F153)XTH21* | 0.167771 | 0.761644 | 0.220275 | 2.13E-23 | 62.429836 |
| *Ac(F153)XTH21/Ac(MD2)XTH23* | 0.167728 | 0.7626 | 0.219942 | 1.99E-23 | 62.508197 |
| *Ac(F153)XTH13/Ac(MD2)XTH14* | 0.273809 | 1.24854 | 0.219304 | 1.01E-20 | 102.33934 |
| *Ac(F153)XTH11/Ac(F153)XTH13* | 0.267844 | 1.27829 | 0.209534 | 2.69E-22 | 104.77787 |
| *Ac(F153)XTH24/Ac(MD2)XTH17* | 0.571834 | 2.7529 | 0.207721 | 3.35E-16 | 225.64754 |
| *Ac(MD2)XTH17/Ac(MD2)XTH19* | 0.326832 | 1.5807 | 0.206764 | 3.96E-22 | 129.56557 |
| *Ac(F153)XTH13/Ac(MD2)XTH13* | 0.267825 | 1.30921 | 0.20457 | 5.18E-23 | 107.3123 |
| *Ac(F153)XTH14/Ac(MD2)XTH14* | 0.354105 | 1.74418 | 0.203021 | 3.30E-22 | 142.96557 |
| *Ac(MD2)XTH11/Ac(MD2)XTH14* | 0.353731 | 1.76402 | 0.200526 | 2.96E-22 | 144.5918 |
| *Ac(F153)XTH18/Ac(MD2)XTH19* | 0.327967 | 1.64334 | 0.199574 | 2.97E-23 | 134.7 |
| *Ac(MD2)XTH14/Ac(MD2)XTH15* | 0.49942 | 2.506 | 0.19929 | 7.32E-18 | 205.40984 |
| *Ac(F153)XTH16/Ac(MD2)XTH14* | 0.49942 | 2.506 | 0.19929 | 7.32E-18 | 205.40984 |
| *Ac(MD2)XTH16/Ac(MD2)XTH19* | 0.335447 | 1.72763 | 0.194166 | 5.13E-23 | 141.60902 |
| *Ac(F153)XTH11/Ac(F153)XTH14* | 0.347583 | 1.81472 | 0.191535 | 9.15E-24 | 148.74754 |
| *Ac(F153)XTH22/Ac(MD2)XTH21* | 0.003968 | 0.0207806 | 0.190949 | 0.0239025 | 1.7033279 |
| *Ac(F153)XTH23/Ac(MD2)XTH21* | 0.003968 | 0.0207806 | 0.190949 | 0.0239025 | 1.7033279 |
| *Ac(F153)XTH11/Ac(MD2)XTH15* | 0.493942 | 2.5892 | 0.190771 | 6.49E-19 | 212.22951 |
| *Ac(F153)XTH14/Ac(MD2)XTH13* | 0.345102 | 1.81631 | 0.190002 | 2.58E-24 | 148.87787 |
| *Ac(F153)XTH11/Ac(MD2)XTH11* | 0.34723 | 1.83593 | 0.18913 | 2.52E-24 | 150.48607 |
| *Ac(MD2)XTH17/Ac(MD2)XTH20* | 0.318924 | 1.69642 | 0.187998 | 2.43E-24 | 139.05082 |
| *Ac(MD2)XTH11/Ac(MD2)XTH13* | 0.344752 | 1.83756 | 0.187614 | 2.37E-24 | 150.61967 |
| *Ac(F153)XTH18/Ac(MD2)XTH20* | 0.320459 | 1.76079 | 0.181998 | 4.00E-25 | 144.32705 |
| *Ac(F153)XTH11/Ac(F153)XTH16* | 0.493942 | 2.78043 | 0.17765 | 2.68E-19 | 227.9041 |
| *Ac(MD2)XTH16/Ac(MD2)XTH20* | 0.327142 | 1.87639 | 0.174347 | 7.28E-25 | 153.80246 |
| *Ac(F153)XTH17/Ac(F153)XTH20* | 0.374812 | 2.15544 | 0.173891 | 3.02E-28 | 176.67541 |
| *Ac(F153)XTH17/Ac(MD2)XTH23* | 0.374699 | 2.16508 | 0.173065 | 1.38E-28 | 177.46557 |
| *Ac(F153)XTH20/Ac(MD2)XTH18* | 0.336175 | 1.99874 | 0.168193 | 1.02E-26 | 163.83115 |
| *Ac(MD2)XTH18/Ac(MD2)XTH23* | 0.336062 | 2.00764 | 0.167391 | 9.89E-27 | 164.56066 |
| *Ac(F153)XTH17/Ac(F153)XTH19* | 0.380575 | 2.32758 | 0.163507 | 4.99E-30 | 190.78525 |
| *Ac(F153)XTH21/Ac(MD2)XTH22* | 0.0039685 | 0.0249975 | 0.158756 | 0.0084384 | 2.0489754 |
| *Ac(F153)XTH17/Ac(MD2)XTH21* | 0.360809 | 2.28707 | 0.157761 | 1.11E-30 | 187.46475 |
| *Ac(F153)XTH9/Ac(MD2)XTH12* | 0.466289 | 2.97165 | 0.156913 | 5.69E-20 | 243.57787 |
| *Ac(F153)XTH9/Ac(MD2)XTH14* | 0.283029 | 1.83311 | 0.154398 | 1.04E-27 | 150.25492 |
| *Ac(F153)XTH9/Ac(F153)XTH11* | 0.289554 | 1.89895 | 0.152481 | 2.54E-28 | 155.65164 |
| *Ac(MD2)XTH18/Ac(MD2)XTH24* | 0.36213 | 2.46754 | 0.146757 | 3.91E-25 | 202.25738 |
| *Ac(F153)XTH17/Ac(MD2)XTH24* | 0.405613 | 2.78448 | 0.145669 | 1.07E-27 | 228.23607 |
| *Ac(F153)XTH17/Ac(MD2)XTH22* | 0.357399 | 2.46159 | 0.14519 | 4.91E-32 | 201.76967 |
| *Ac(MD2)XTH13/Ac(MD2)XTH15* | 0.490935 | 3.44764 | 0.142397 | 1.05E-20 | 282.59344 |
| *Ac(F153)XTH9/Ac(MD2)XTH13* | 0.289532 | 2.05299 | 0.141029 | 7.86E-30 | 168.27787 |
| *Ac(MD2)XTH10/Ac(MD2)XTH14* | 0.246789 | 1.78673 | 0.138124 | 7.25E-29 | 146.45328 |
| *Ac(F153)XTH11/Ac(MD2)XTH10* | 0.254546 | 1.85506 | 0.137217 | 1.93E-29 | 152.0541 |
| *Ac(MD2)XTH9/Ac(MD2)XTH14* | 0.246789 | 1.85584 | 0.13298 | 1.79E-29 | 152.11803 |
| *Ac(MD2)XTH18/Ac(MD2)XTH21* | 0.326011 | 2.46732 | 0.132132 | 1.19E-30 | 202.23934 |
| *Ac(F153)XTH10/Ac(MD2)XTH14* | 0.263186 | 2.02829 | 0.129758 | 2.41E-31 | 166.25328 |
| *Ac(F153)XTH10/Ac(F153)XTH11* | 0.270236 | 2.10716 | 0.128247 | 6.32E-32 | 172.71803 |
| *Ac(F153)XTH11/Ac(MD2)XTH9* | 0.254546 | 2.00913 | 0.126695 | 7.30E-31 | 164.68279 |
| *Ac(MD2)XTH10/Ac(MD2)XTH13* | 0.254527 | 2.01141 | 0.126542 | 7.05E-31 | 164.86967 |
| *Ac(F153)XTH10/Ac(MD2)XTH13* | 0.270216 | 2.32074 | 0.116435 | 2.18E-33 | 190.22459 |
| *Ac(MD2)XTH9/Ac(MD2)XTH13* | 0.254527 | 2.20625 | 0.115366 | 4.04E-32 | 180.84016 |
| *Ac(MD2)XTH18/Ac(MD2)XTH22* | 0.323584 | 2.81778 | 0.114836 | 1.21E-32 | 230.96557 |
| *Ac(F153)XTH16/Ac(MD2)XTH13* | 0.490935 | 4.36862 | 0.112377 | 4.12E-21 | 358.08361 |
| *Ac(F153)XTH19/Ac(MD2)XTH18* | 0.353241 | 3.20272 | 0.110294 | 3.71E-31 | 262.51803 |
| *Ac(F153)XTH12/Ac(MD2)XTH14* | 0.278271 | 2.65352 | 0.104869 | 3.14E-34 | 217.50164 |
| *Ac(MD2)XTH18/Ac(MD2)XTH19* | 0.348627 | 3.3488 | 0.104105 | 1.01E-29 | 274.4918 |
| *Ac(F153)XTH17/Ac(MD2)XTH19* | 0.348627 | 3.3488 | 0.104105 | 1.01E-29 | 274.4918 |
| *Ac(F153)XTH7/Ac(MD2)XTH6* | 0.0030774 | 0.0300402 | 0.102442 | 0.0030984 | 2.4623115 |
| *Ac(MD2)XTH18/Ac(MD2)XTH20* | 0.345619 | 3.43024 | 0.100757 | 1.98E-30 | 281.16721 |
| *Ac(F153)XTH17/Ac(MD2)XTH20* | 0.345619 | 3.43024 | 0.100757 | 1.98E-30 | 281.16721 |
| *Ac(F153)XTH17/Ac(MD2)XTH16* | 0.310563 | 3.18529 | 0.0974991 | 1.03E-39 | 261.08934 |
| *Ac(F153)XTH11/Ac(F153)XTH12* | 0.274586 | 3.11901 | 0.088036 | 2.38E-37 | 255.65656 |
| *Ac(F153)XTH22/Ac(MD2)XTH22* | 0.0013199 | 0.0165958 | 0.0795325 | 0.0130468 | 1.3603115 |
| *Ac(F153)XTH23/Ac(MD2)XTH22* | 0.0013199 | 0.0165958 | 0.0795325 | 0.0130468 | 1.3603115 |
| *Ac(F153)XTH17/Ac(F153)XTH18* | 0.304512 | 3.83331 | 0.0794383 | 2.18E-42 | 314.20574 |
| *Ac(F153)XTH12/Ac(MD2)XTH13* | 0.274936 | 3.48501 | 0.078891 | 8.18E-38 | 285.65656 |
| *Ac(F153)XTH3/Ac(MD2)XTH4* | 0.0752726 | 0.962535 | 0.0782025 | 6.96E-44 | 78.896311 |
| *Ac(F153)XTH2/Ac(F153)XTH3* | 0.0752726 | 0.962535 | 0.0782025 | 6.96E-44 | 78.896311 |
| *Ac(F153)XTH14/Ac(MD2)XTH11* | 0.0030821 | 0.0420219 | 0.0733454 | 0.0002119 | 3.444418 |
| *Ac(F153)XTH17/Ac(F153)XTH22* | 0.34941 | 5.10982 | 0.06838 | 4.29E-37 | 418.8377 |
| *Ac(F153)XTH17/Ac(F153)XTH23* | 0.34941 | 5.10982 | 0.06838 | 4.29E-37 | 418.8377 |
| *Ac(MD2)XTH2/Ac(MD2)XTH4* | 0.0608331 | 0.9458 | 0.0643192 | 4.83E-45 | 77.52459 |
| *Ac(F153)XTH2/Ac(MD2)XTH2* | 0.0608331 | 0.9458 | 0.0643192 | 4.83E-45 | 77.52459 |
| *Ac(F153)XTH3/Ac(MD2)XTH3* | 0.0579823 | 0.945127 | 0.0613487 | 6.26E-45 | 77.469426 |
| *Ac(MD2)XTH2/Ac(MD2)XTH3* | 0.0565882 | 0.961932 | 0.0588276 | 3.14E-46 | 78.846885 |
| *Ac(F153)XTH18/Ac(MD2)XTH18* | 0.265729 | 4.64275 | 0.0572353 | 1.19E-40 | 380.55328 |
| *Ac(MD2)XTH16/Ac(MD2)XTH18* | 0.260828 | 4.77968 | 0.0545702 | 2.00E-40 | 391.77705 |
| *Ac(F153)XTH11/Ac(MD2)XTH13* | 0.0015303 | 0.0324607 | 0.0471433 | 0.00065 | 2.6607131 |
| *Ac(MD2)XTH13/Ac(MD2)XTH14* | 0.0015797 | 0.0394178 | 0.0400752 | 0.0001589 | 3.2309672 |
| *Ac(MD2)XTH9/Ac(MD2)XTH10* | NA | 0.0174331 | 0 | NA | 1.4289426 |
| *Ac(F153)XTH9/Ac(MD2)XTH9* | NA | 0.0174331 | 0 | NA | 1.4289426 |
| *Ac(F153)XTH9/Ac(MD2)XTH10* | NA | 0.011577 | 0 | NA | 0.9489344 |
| *Ac(F153)XTH16/Ac(MD2)XTH15* | NA | 0.0051028 | 0 | NA | 0.4182656 |
| *Ac(F153)XTH11/Ac(MD2)XTH14* | NA | 0.0055047 | 0 | NA | 0.4512033 |
