# Supplementary material for: Genome-Wide Identification and Characterization of Xyloglucan Endotransglycosylase/Hydrolase in Ananas comosus during Development
Source: Genes (Basel). 2019 Jul 16;10(7):537. doi: 10.3390/genes10070537 (PMC6678617; doi:10.3390/genes10070537)
Supplement: Supplementary file 1 [file genes-10-00537-s001.zip › Supplementfiles/File1.pdf]

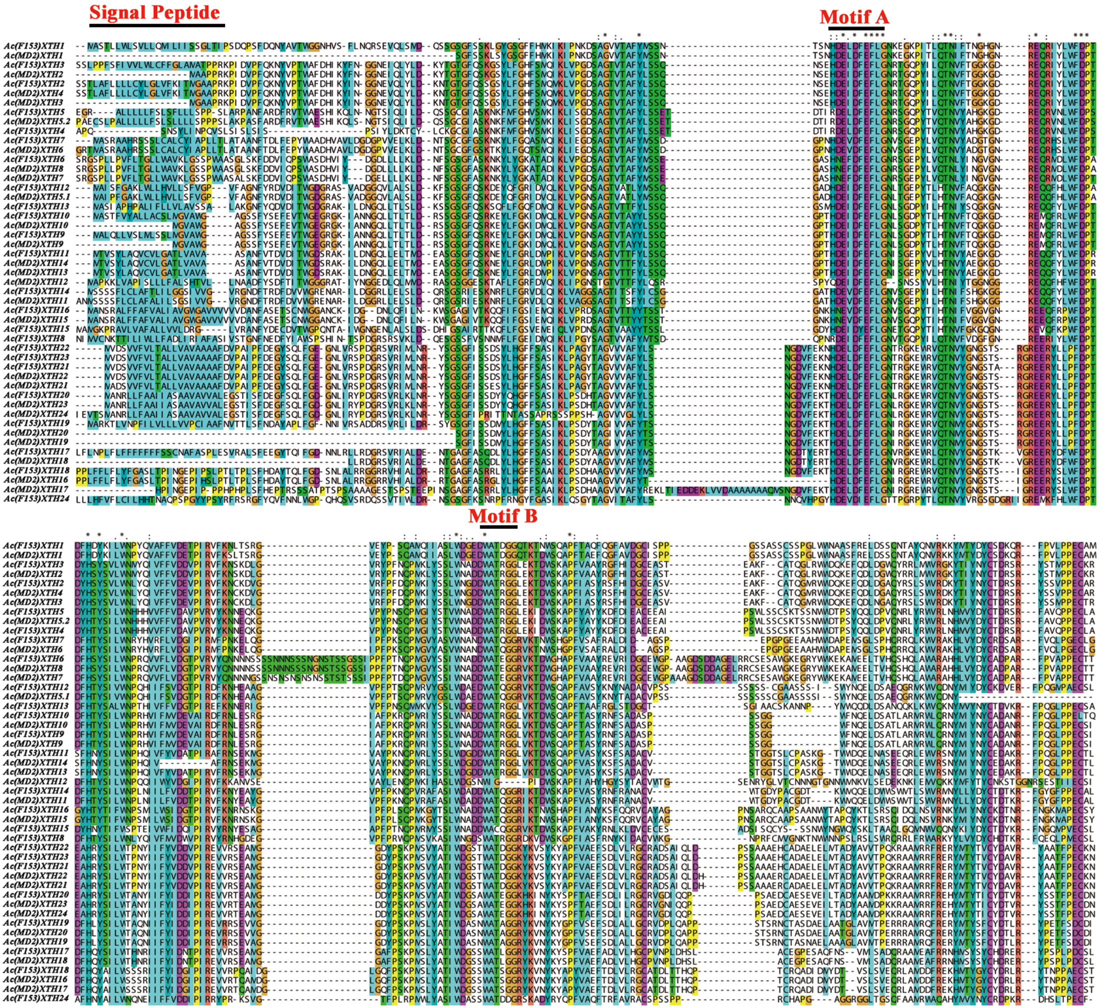
Signal Peptide
Motif A
Motif B
