# Supplementary figures and images for: Genome-Wide Identification and Characterization of Xyloglucan Endotransglycosylase/Hydrolase in Ananas comosus during Development
Source: Genes (Basel). 2019 Jul 16;10(7):537. doi: 10.3390/genes10070537 (PMC6678617; doi:10.3390/genes10070537)

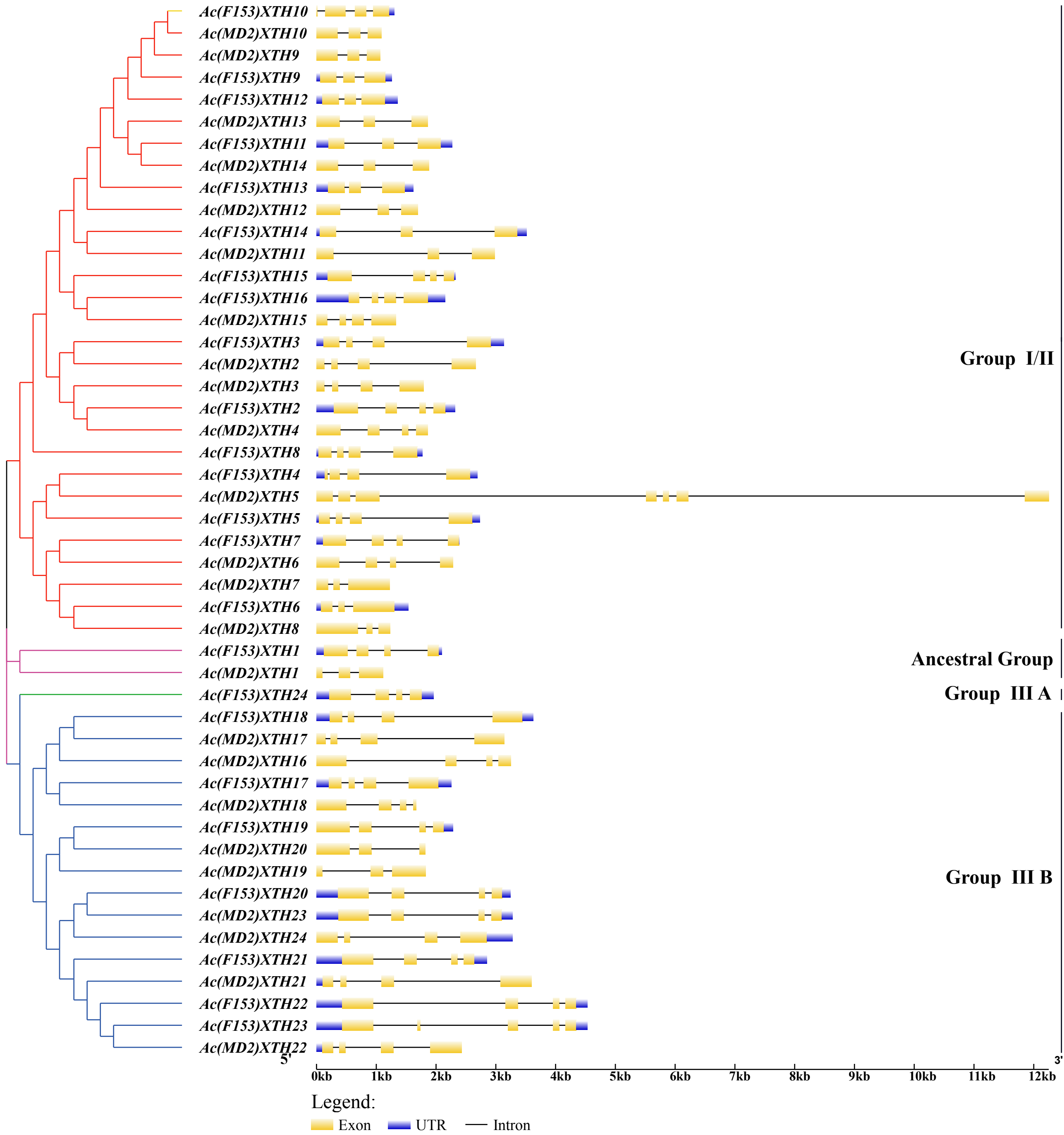

Supplement: Supplementary file 1 [file genes-10-00537-s001.zip › Supplementfiles/File2.pdf]

**A****Relative expression levels***Ac(MD2)XTH18*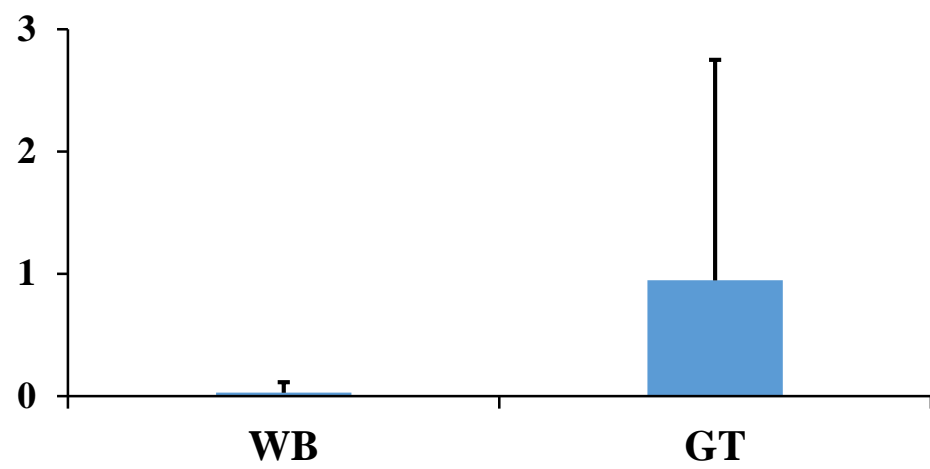*Ac(MD2)XTH11*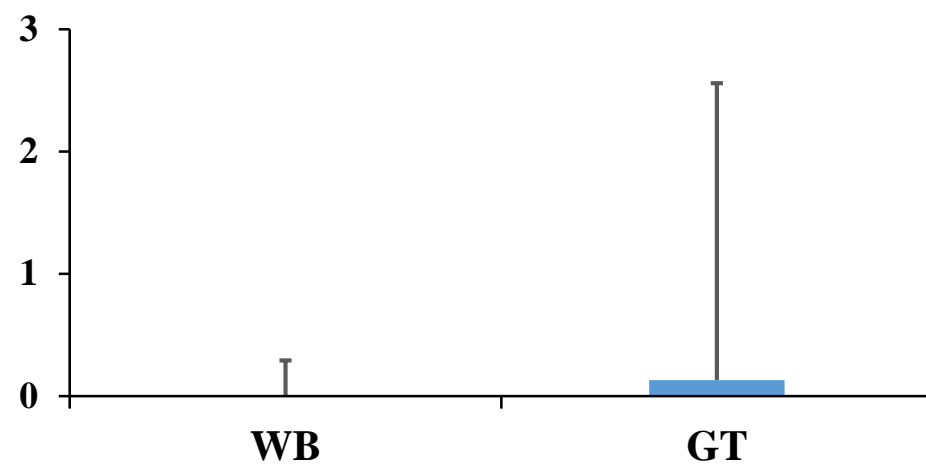**B****Relative expression levels****WB**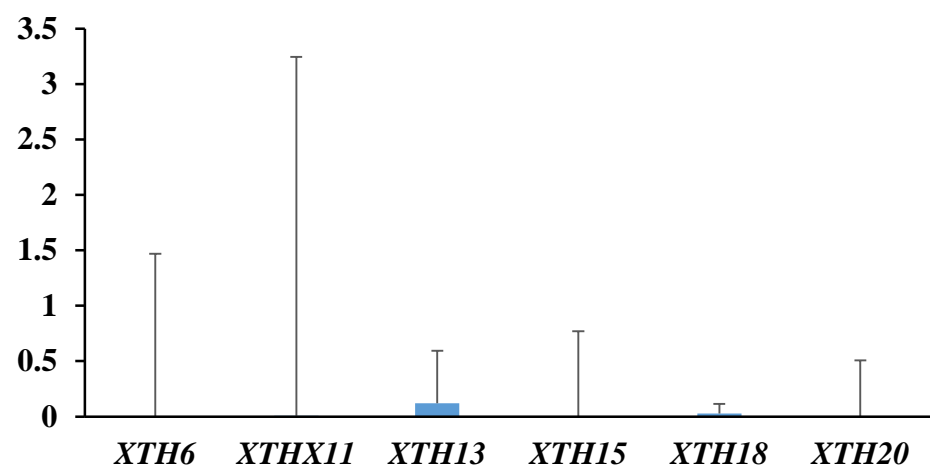

Supplement: Supplementary file 1 [file genes-10-00537-s001.zip › Supplementfiles/File3.pdf]

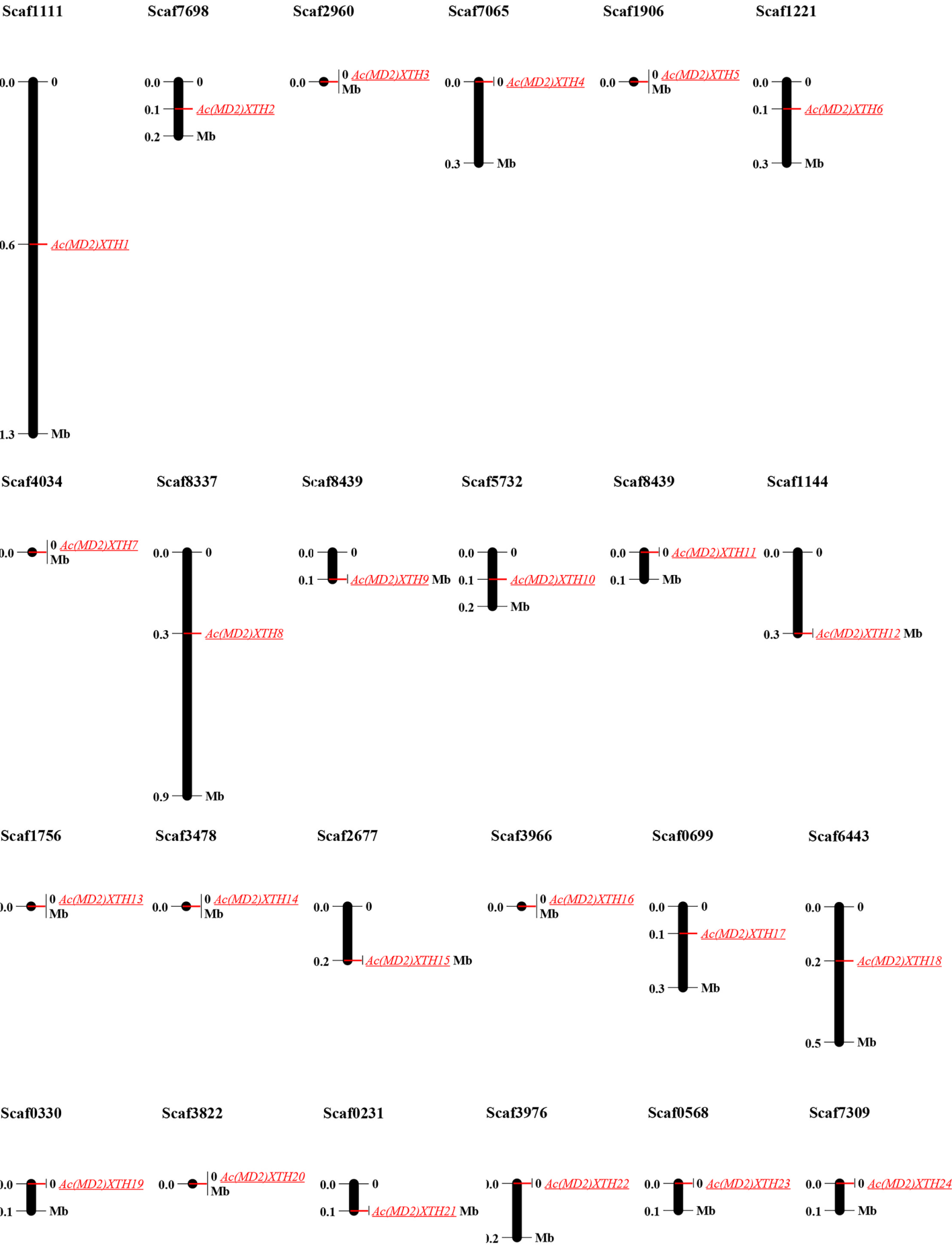

Supplement: Supplementary file 1 [file genes-10-00537-s001.zip › Supplementfiles/File4.pdf]
